# Optogenetics for Investigating and Targeting Hallmark Traits of Cancer

**DOI:** 10.3390/biom16020217

**Published:** 2026-02-02

**Authors:** Hannah Kienbacher, Muhammad Hashim, Michael Grusch

**Affiliations:** 1 Center for Cancer Research, Comprehensive Cancer Center Vienna, Medical University of Vienna, 1090 Vienna, Austria; hannah.kienbacher@edu.kl.ac.at (H.K.); mhashim@bs.qau.edu.pk (M.H.); 2 Karl Landsteiner University of Health Sciences, Krems Campus, Dr.-Karl-Dorrek-Straße 30, 3500 Krems, Austria

**Keywords:** optogenetics, cancer research, signal transduction, immune cell modulation, cancer therapy

## Abstract

The light-mediated, specific, and precise control of cell functions enabled by optogenetics has become a versatile method for investigating and combatting cancer. An increasing set of optogenetic tools enables tightly controlled regulation of ion flux across biological membranes, gene expression, gene editing, and protein–protein interactions and is being used to interrogate hallmark traits of cancer at the cellular, subcellular, and organismic level. This enables, on the one hand, the identification of critical signaling circuits required for cancer development and progression in vitro and in animal models and can flag potential intervention points for pharmacologic interference. On the other hand, optogenetics can improve the level of control in cell-based therapeutics. The current article provides a review of optogenetic tools and approaches used in the cancer research field and their multiple applications for improving our understanding of signal transduction pathways, modulating immune functions in the tumor microenvironment, facilitating drug screening, or directly attacking cancer cells. Key advantages and achievements of optogenetics in the cancer research field and remaining barriers for clinical applications are discussed.

## 1. Introduction and a Glimpse of History

Optogenetics is a biological approach that combines optical and genetic methods to control cell functions. The technology typically involves artificially incorporating DNA sequences that encode light-sensitive proteins or protein domains, which are often derived from plants or microorganisms, into selected cells or organisms [1,2]. Once the encoded proteins are expressed in the receiving cells, their activity can be controlled with light, which in turn enables a light-mediated control of a wide spectrum of specific cell functions depending on the engineered proteins. DNA constructs are delivered using established transfection techniques including plasmids, viral vectors, or bacterial carriers [3]. Optogenetic proteins can, for instance, function as ion channels, cell surface receptors, or various components of cell signaling cascades [4].

Optogenetics originated in the field of neuroscience in response to a critical need for selective control of individual neurons while leaving surrounding neurons unaltered. Previously thought to be unattainable, a 2005 article demonstrated that neurons could be engineered to respond to light upon the integration of microbial opsin genes without the need for additional components [1,5]. In subsequent years, optogenetics has become a powerful tool to dissect physiological and pathophysiological neuronal circuits and processes [1,6]. Specifically, the modulation of neuronal behavior is achieved by altering membrane potential using photoreceptive proteins including bacteriorhodopsin (BR), halorhodopsin (NpHR), and channelrhodopsin-2 (ChR2) [7]. Further developments in optogenetics have expanded the toolbox with the introduction of additional light-sensitive proteins or protein domains such as phytochromes (PHY), cryptochromes (CRYs), and light–oxygen–voltage (LOV) domains [8,9]. These light-sensitive protein domains have been coupled to various effector proteins involved in many different signaling pathways to enable light-mediated regulation of the respective pathways [10,11,12]. Consequently, optogenetics has expanded beyond neuroscience into various scientific fields and also found applications in cancer research and translational oncology [4,13,14].

## 2. Key Features and Advantages of Optogenetics

One key advantage of integrating photoreceptors into specific cells within living tissues is the ability to control cellular events within milliseconds. This capability enables researchers to observe real-time changes and effects within complex networks [1]. While cancer development overall is a process taking years rather than milliseconds, the ability to interrogate distinct signaling events in real-time is often fundamental for understanding their contribution to the process. Another key advantage is the non-invasive nature of stimulation, which allows repeated activation and withdrawal of the stimulus without affecting cells that do not express the optogenetic construct [15]. The third critical advantage is the high spatial precision that is achievable by directing illumination only to individual cells or even organelles and cellular subcompartments [16]. Thus, light serves as a remote control providing specific and spatiotemporally precise activation and deactivation of cellular functions in intact cells or organisms with minimal cytotoxicity [17,18].

**Figure 1:**
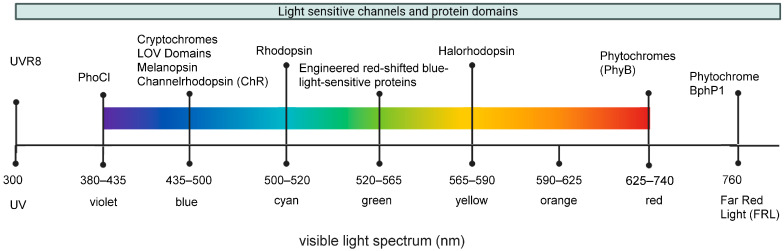
Distribution of light-responsive proteins and protein domains commonly used in optogenetic tools across the electromagnetic spectrum.

Continuous advancements in the development of optogenetic proteins have expanded the available tools, which now contain proteins that can be activated with light of different wavelengths (Figure 1) providing additional experimental flexibility and enabling multiplexing by combination of several optogenetic proteins activated simultaneously or with alternating patterns [4,16,19]. Different light-sensitive proteins and protein domains do not only show different spectral sensitivities but also different activation and inactivation kinetics and modes of action as shown in Table 1 for representative photoreceptors used in optogenetic tools. These characteristics of light-responsive protein domains are key features for selecting appropriate optogenetic tools to answer specific research questions. For potential therapeutic applications in the cancer field, the versatility of optogenetic systems allows them to be effectively combined with other technologies such as gene therapies [20], bacteria- or virus-mediated therapies [20,21,22,23], photodynamic therapy [24] and, in particular, different forms of immunotherapy [13,25,26].

## 3. The Optogenetic Toolbox for Cancer Research

**Figure 2:**
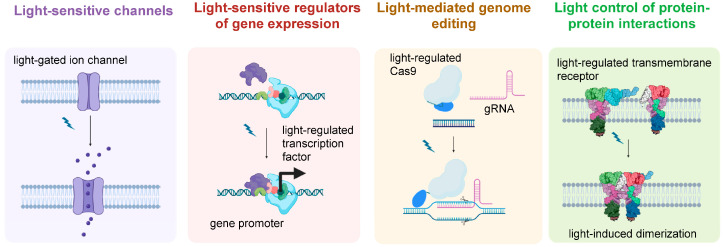
Optogenetic tools applied in cancer research can be broadly categorized into light-sensitive channels, light-responsive regulators of gene expression, light-responsive tools for gene editing, and light-mediated regulators of protein localization and protein–protein interactions. The depicted examples show the light-mediated opening of an ion channel, the light-dependent activation of a gene promoter, the light-controlled DNA cleavage by Cas9, and the light-induced dimerization of a transmembrane receptor, respectively.

Since its early days, an increasing number of proteins and protein domains from all kingdoms of life were genetically fused to a plethora of different unrelated protein domains in order to achieve light-regulated control over a wide range of cellular functions. In the majority of optogenetic systems, light results in the activation of a specific signal; however, tools for light-mediated signal inhibition and gene disruption have also been designed. Moreover, while some optogenetic systems have been engineered to induce, for instance, rapid and spatially confined but irreversible effects, the usefulness of others lies in their ability to allow repeated switching between two states. Several excellent review articles have described the activation and inactivation mechanisms of a broad range of optogenetic proteins in detail [9,11,12]. With respect to their use in cancer research, light-regulated actuators can broadly be divided into light-regulated channels, light-sensitive regulators of gene expression, light-sensitive regulators of protein localization and protein–protein interactions, and tools for light-mediated gene editing (Figure 2).

### 3.1. Light-Sensitive Channels

Channelrhodopsin (ChR) is a common component of optogenetic systems, as it mediates cation flow across cell membranes upon blue light illumination, thereby altering membrane potential. It was originally isolated from *Chlamydomonas reinhardtii*, a green freshwater alga [36,37]. As channelrhodopsin shows favorable optogenetic characteristics, many variants (e.g., ChETA, CatCH) have been constructed, showing different cation affinity and channel kinetics [36,38,39,40,41]. ChR and its variants have been used for applications as diverse as inducing cell death [39,42], exploring and modulating the tumor microenvironment [43,44], altering T cell activity [45,46], and modulating Ras function [40]. In contrast to ChR, halorhodopsin and bacteriorhodopsin have been used in only a few cancer research-related studies, for instance, to investigate calcium-regulated cell migration [47] and cell proliferation in lung cancer cells [48].

### 3.2. Light-Sensitive Regulators of Gene Expression

A number of different synthetic transcription factor/promoter systems for light-regulated gene expression in mammalian cells and bacteria have been developed [49,50]. Applications of the blue light-sensitive promoter pDawn [51,52,53,54], photosensitive bacterial transcription factor EL222 [22,23], and the HSP70 promoter [55,56,57] have demonstrated their potential for bacteria-mediated cancer therapies, immunotherapies, and control of local gene expression. Moreover, light-controlled, engineered Clustered Regularly Interspaced Palindromic Repeat (CRISPR)-Cas systems have been developed [58] and deployed for gene activation of endogenous promoters as gene therapy approaches [20,59,60]. Another system, based on CRISPR/Cas13b was developed for light-induced RNA knockdown [61]. A different RNA knockdown system uses near-infrared (NIR) light to activate the HSP70 promoter for expression of a gene-silencing construct against human telomerase [56].

An alternative approach for rapid control of protein expression levels is to alter protein degradation. One recently developed system uses a light-controlled version of TRIM21, an E3 ubiquitin ligase that can target proteins bound to specific peptides for proteosomal degradation [62]. Using light-controlled TRIM21 in combination with peptides binding the key signaling protein phosphoinositide 3-kinase (PI3K) and the ferroptosis regulator glutathione peroxidase 4 (GPX4) resulted in their rapid light-induced degradation.

### 3.3. Light-Mediated Genome Editing

Since cancer is a disease characterized by recurrent mutations and gene deletions, gene knock-out and gene editing technologies have been extensively used to understand cancer development and therapeutic vulnerabilities of cancer. Optogenetic genome editing tools offer the potential to achieve precise and spatially confined genetic alterations in a non-invasive or minimally invasive way. For that purpose, light-controlled CRISP-Cas genome editing systems have been developed and used in cancer cell lines and xenograft models [24,63,64].

Long before the arrival of CRISPR-Cas9 technology, Cre recombinase systems have facilitated genetic engineering. To increase the versatility of the Cre-lox system, light-regulated derivatives of Cre have been engineered, combined with the more traditional regulation of Cre activity by tamoxifen [65] and doxocyclin [66], and used for in vivo tracing experiments [66] to investigate malignant transformation at the single-cell level [67], as well as to enhance the level of spatiotemporal control in chimeric antigen receptor (CAR)-T cell immunotherapeutic approaches [65].

### 3.4. Light Control of Protein Localization and Protein–Protein Interactions

Perhaps the most widespread application of optogenetic systems in cancer research is the light-regulated control over various forms of protein–protein interactions that control the activity or subcellular localization of proteins involved in cancer development and therapy.

One such approach adds light-sensitive domains to a target protein and thereby the protein remains caged and in an inactive state until illumination triggers a conformational change to release the protein, enabling interaction with its binding partners [37]. Such systems have, for instance, been applied to control the nuclear localization of proteins by using the light–oxygen–voltage domain 2 of *Avena sativa* phototropin 1 (ASLOV2) to conceal (in the dark) or present (in response to blue light illumination) a nuclear localization sequence (NLS) to the importins of the cell [68].

Many physiological cell functions depend on dimerization or oligomerization of signaling proteins. Therefore, several light-regulated systems for homo- or heterodimerization and oligomerization have been designed using, for instance, LOV domains of various origin or cryptochrome 2 (CRY2) or phytochrome-B (PhyB) of *Arabidopsis thaliana* and their respective binding partners (CIB and PIF, respectively) [69]. These tools have been used to control the activity of key cancer-related growth factor receptors such as the epidermal growth factor receptor (EGFR) or receptors of the transforming growth factor beta (TGF-beta) family [62,70,71,72] as well as intracellular signaling molecules including B-Raf and PI3K [73,74].

An alternative approach to control the activity or localization of a cellular protein without engineering the endogenous protein itself, is the use of light-switchable engineered nanobodies. Nanobodies are derived from the single variable domain of the antibodies of camelids and like conventional antibodies can be produced as high affinity binders against a high number of specific target proteins [75]. Inserting a LOV domain enables a light-regulated control over target binding affinity, thereby altering the activity or localization of the target protein [76]. Related approaches have engineered other forms of light-switchable intracellular antibodies (intrabodies) and synthetic non-immunoglobin protein binders (monobodies) that could potentially be used to control a broad range of cancer-relevant signaling molecules [77,78,79].

## 4. Applications of Optogenetics in Cancer Research and Translational Oncology

Optogenetic tools and approaches have been successfully applied in several key areas of cancer research and tested for their applicability in various preclinical models with the aim of studying cell signaling, modulating immune functions, facilitating drug screening, or eliminating malignant cells.

### 4.1. Dissecting Signaling Pathways

**Figure 3:**
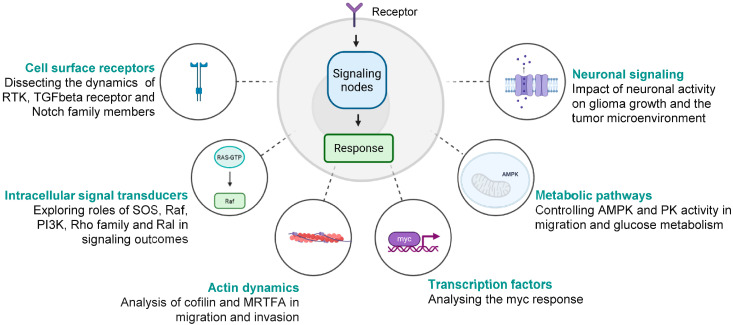
Examples of signal transduction pathway analyses at different levels—from the cell surface to the cell nucleus—that have been enabled or facilitated by optogenetic approaches.

The most distinct feature of malignant cells is their ability to chronically sustain growth, promoting signaling and acquiring greater autonomy over the cell cycle, angiogenesis, apoptosis regulation, migration, and invasion [80]. Light-controlled receptors and downstream signaling molecules offer the potential to dissect the underlying signaling dynamics with spatial and temporal precision and thereby improve our understanding of cancer development and flagging key signaling nodes that offer the most effective therapeutic interventions. Within the last decade, optogenetic tools to interrogate almost all relevant signal transduction pathways have been generated and used to dissect various aspects of the behavior of malignant cells. Figure 3 highlights examples of the investigation of cell signaling mechanisms with optogenetics at various levels.

#### 4.1.1. Receptor Tyrosine Kinases

Receptor tyrosine kinases (RTKs) are a family of transmembrane cell surface receptors, that form homo- or heterodimers after the binding of growth factors (GFs) or hormones [81]. This receptor family is involved in a variety of physiological and pathological cellular behaviors like proliferation, migration, and epithelial–mesenchymal transition (EMT). The generation of the first light-controlled RTKs (Opto-RTKs) using receptor homodimerization with blue light [70,82] was rapidly followed by various other systems enabling homo- and heterodimerization of receptors with light of different wavelengths [83,84]. These approaches have enabled the regulated activation of key oncogenic RTKs such as the epidermal growth factor receptor (EGFR), vascular endothelial growth factor receptor (VEGFR), fibroblast growth factor receptor 1 (FGFR1), proto-oncogene rearranged during transfection (RET) and the neurotrophin receptors TrkA/B/C. Together with optogenetically engineered downstream proteins, like the guanine nucleotide exchange factor (GEF) son of sevenless (SOS) and signaling proteins involved in the mitogen-activated protein kinase (MAPK) and phosphoinositide-3-kinase (PI3K)/Akt pathway (see below), light control of RTKs can, for instance, help to more precisely define the role of RTKs in processes such as cell migration and EMT, key functionalities in cancer progression and metastasis.

#### 4.1.2. TGF-Beta Signaling

Members of the TGF-beta receptor family are activated by TGF-betas, activins, bone morphogenetic proteins (BMP), and growth and differentiation factors (GDF) [85]. They play important roles in development, differentiation, and the regulation of cell death. In cancer, they can have both tumor suppressive and oncogenic functions. TGF-beta 1 is one of the most potent inducers of EMT and has strong immunosuppressive functions [86]. Optical control of TGF-beta signaling has been used to investigate the dynamics of nuclear translocation of downstream Smad proteins [71] and to demonstrate the contribution of the TGF-beta family receptor ACVR1 to TGF-beta-induced SMAD1/5 pathway activation and EMT induction [87]. Optical activation of BMP signaling has been described [88] but not yet used for investigating BMP signals in cancer.

#### 4.1.3. Notch Signaling

The Notch receptors represent another type of transmembrane receptors, in which the intracellular domains act as transcription factors after proteolytic cleavage upon stimulatory ligand binding [89]. Notch target genes are, for instance, Myc and the Hey family (HEY/HES). Notch signaling is involved in physiological processes such as mechanosensing and differentiation and has been associated, especially in breast cancers, with metastasis, cell migration, and chemoresistance [90,91]. In estrogen-positive MCF7 and triple-negative MDA-MV-468 breast cancer cell lines, an Opto-Notch construct was stably expressed and confirmed a correlation between Notch activity and oncogenic breast cancer proliferation and chemoresistance [91].

#### 4.1.4. Ras–Raf–Mek–Erk Signaling

After extracellular stimulation of cell surface receptors, corresponding induction of cellular events happens through complex and intertwined signaling networks [92]. The mitogen-activated protein kinase (MAPK) pathway transmits signals through the activation of small G proteins (Ras) and a cascade of protein kinases (Raf–Mek–Erk). Furthermore, it regulates different cellular processes like cell differentiation, proliferation, and cell cycle control [93]. Ras to Erk signaling kinetics determine the ability of a cell for appropriate decision-making [94,95]. Perturbations of transmission dynamics caused by endogenous (mutations, changes in the cellular microenvironment) or exogenous (small molecule inhibitors (SMIs)) stimuli can result in a corrupted signal perception of cells, ultimately leading to disease [94].

The optogenetic activation of C-Raf [73,92] or B-Raf [96], as well as OptoSOS [94], can be utilized to investigate MAPK signal transmission kinetics. Disrupted surveillance mechanism balancing cell fate [73] led to changes in the transcription machinery and increased proliferation within normally non-proliferative Ras transmission patterns [94]. Diminished MAPK signaling dynamics, such as those induced by DNA damage, correlate with prolonged signal activity [73]. Thus, genetic damage prolongs the usual pulsatility of MAPK signals, consequently attenuating the G2 checkpoint and fostering mitotic entry, cellular transformation, and genomic instability [73].

Likewise, frequent mutations in B-Raf [92] and the application of small molecule inhibitors targeting Raf [96], lead to disturbances in MAPK signaling kinetics. Increased B-Raf homo- or heterodimerization, and paradoxical C-Raf activation with subsequent Erk augmentation promote tumorigenesis [92]. In vivo zebrafish experiments showed that transient KRasG12V expression at an early stage of embryogenesis and constitutive expression in adult fish increased the probability of tumor formation [97]. Therefore, Optogenetics can help decoding mechanisms behind paradoxical C-Raf activation and consequences of changes in MAPK signaling frequency [92].

#### 4.1.5. PI3K Signaling

Apart from the initiation of MAPK signaling, RTK and Ras activity can also lead to PI3K activation and downstream signaling via the effector proteins Akt and mammalian target of rapamycin (mTOR) [98]. Since hyperactivation of this signaling network is a frequent feature of malignancies associated with metastasis, augmented proliferation, and cytostatic drug resistance, multiple approaches to optically control the PI3K/Akt/mTor pathway have been made [74,99,100,101,102]. One such system termed PPAP2 was used to elucidate the effects of PI3K hyperactivation in cancer cells and identified an increased amount of anti-apoptotic tumor necrosis factor alpha-induced protein 8 (TNFAIP8) due to a PI3K-dependent upregulation in translation of preexisting messenger ribonucleic acid (mRNA) [74]. This mechanism enables cells to evade cell death induced by alkylating agents. These results suggested that targeting TNFAIP8 appears to be more beneficial than using mTor as a cancer target, as the latter also executes other physiological functions [74]. Thus, decoding PI3K-dependent mechanisms behind drug resistance with the help of optogenetic tools could help to overcome challenges in cancer treatment by developing new beneficial combination therapies [74,101].

Next to TGF-beta signaling, the PI3K/Akt/mTor plays a vital role in the process of EMT [103]. As tumor metastasis relies on the migratory capability of mesenchymal cells, initiation and promotion of metastasis is determined by the EMT process. In vitro studies with A549 cells have shown that specific and reversible optical activation of PI3K leads to the downregulation of the epithelial cell adhesion molecule E-Cadherin, which is closely associated with the EMT process [99].

#### 4.1.6. The Myc Oncogene

Myc (c-Myc) is a transcription factor that responds to the growth factor and mitogen activation and is frequently deregulated and overexpressed in different forms of cancer [104]. Investigating its role with single-cell imaging and optogenetics has suggested that Myc increases the active time of genes rather than augmenting their activation frequency [105]. The data suggest that Myc leads to an increased duration of transcriptional bursts by altering the binding dynamics of transcription factors and thereby exerts global changes in the dynamics of the transcription machinery.

#### 4.1.7. RhoGTPase Family

Migration and cancer cell motility are based on actin-protrusions, like non-apoptotic membrane blebs, filopodia, and lamellipodia. These projections are associated with the small GTPases Rho, Rac, and Cdc42, which can function as oncogenes affecting cytoskeletal dynamics by acting as molecular switches [106]. The specific and spatial control of RhoA with different optogenetic systems has been used to study its role in the cell dissociation of collectively migrating cancer cell sheets [107], amoeboid migration [108], and control of the direction in cell locomotion [109], as well as in cytokinesis [110].

Optogenetic activation of Cdc42 was used together with an optogenetic RhoA construct in analyzing the regulation of migration direction and in filopodia formation [109,111]. Reversible light-induced stimulation of photoactivatable Rac1 resulted in lamellipodia formation in prostate cancer cells and was used to dissect the role of PI3K signaling in lamellipodia formation and ruffling [112]. Not only lamellipodia, but also adherens-junctions (AJs) are controlled by Rac1, as an optogenetic stimulation of Rac1 was shown to drive junctional F-actin assembly via Formin-like 2 (FMNL2) [113]. Furthermore, optogenetic activation of Rac1 was used to demonstrate that spatially localized protrusions induced by Rac1 can drive Erk activation [114].

Overall, optogenetic tools controlling the activity of RhoGTPase family members have been instrumental for elucidating their respective spatially and temporally confined contributions to cell migration, cytokinesis, and EMT.

#### 4.1.8. RalGTPase, Myocardin-Related Transcription Factor A, and Cofilin Signaling

RalA and RalB are two additional members of the Ras family of GTPases that act downstream of Ras oncoproteins in the regulation of cell survival and metastasis [115]. Optical stimulation demonstrated that localized RalB activation, particularly at cell edges, heightened cellular dynamics resulting in the formation of protrusions independent of Rac1 and thereby enhanced motile and invasive abilities [116]. Likewise, myocardin-related transcription factor A (MRTFA) unveiled its role in actin dynamics, tumor cell invasion, and migration, as well as in the formation of membrane blebs when investigated with the help of a light-controlled construct [117].

Two different optogenetic strategies were employed to investigate cofilin, an actin remodeling protein [118]. In one study, light-mediated control of nuclear versus cytoplasmic distribution of cofilin was used to investigate chromatin organization in the cell nucleus at the exit of mitosis [119]. Another study employed a strategy, in which intramolecular light-modulated bridges block active sites in the inactivated state and reveal them upon light irradiation. This was used to investigate the role of cofilin in the formation of invadopodia, cell protrusions enabling the degradation of the cell matrix thereby facilitating the process of tumor cell invasion into tissues [120].

#### 4.1.9. Impact of Membrane Potential on Migration Ability

Intracellular calcium (Ca^2+^) concentration is tightly controlled, given its critical involvement in the modulation of signaling pathways that mediate processes like contraction, cell death, gene transcription, proliferation, focal adhesion, migration, and cytoskeletal remodeling [121]. Alterations in the constitutive calcium entry (CCE), which is regulated by transient receptor potential (TRP) ion channels, is closely associated with pathological conditions [122]. In non-excitable mouse myoblast cells, halorhodopsin (eNpHR), a photoactivatable chloride pump, was employed to spatially and temporally activate CCE. Light-regulated alterations in the membrane potential of cells led to changes in cell migration via calcium entry mediated by TRP vanilloid 2 (TRPV2) channels [47].

#### 4.1.10. Metabolic Pathways

Together with other technologies, optogenetics established a critical role of mitochondria in metastasis of ovarian carcinoma cells [123]. Cytoskeletal dynamics at the leading edges of migrating cells utilize adenosine triphosphate (ATP) at high rates, therefore promoting the activation of adenosine monophosphate (AMP)-activated protein kinase (AMPK) locally. AMPK is a crucial sensor of cellular energy balance and recruits mitochondria into lamellipodia at the front end of cells supporting protrusion formation through locally synthesized ATP. Microtubule-based mitochondrial trajectories exhibit polarization, regulation, and direction, rather than displaying stochastic activity. Although, many cancer cells show a Warburg-effect (shift to preferential use of aerobic glycolysis for energy production), subcellular reversals of this phenomenon occur at the leading edge. Mitochondrial ATP synthesis in protrusive structures established during chemotaxis is the main driving force of energy production. Creating a photo-switchable AMPK-inhibitory peptide, the vital function of AMPK in membrane ruffling for migration and invasion of malignant cells in a mitochondria-dependent manner was demonstrated [123].

Malignancies exemplify how alterations in the allosteric regulation of proteins involved in metabolism can lead to pathologies. In the glucose metabolism, which is particularly vital in many tumors, pyruvate kinases (PK) are involved in the process of converting glucose into pyruvate and ATP [124]. Especially the PK M2 is commonly expressed in tumors, as well as in normal cells, and is allosterically activated among others by fructose 1,6-bisphosphat (FBP). In order to model allosteric regulation, a version of PK M2 was generated that allows a light-mediated rapid and remote-controlled activation of PK activity [124].

#### 4.1.11. Neuronal Signaling in Cancer Progression

Optogenetic experiments harboring the opsins ChR2 [43,44,125,126] and ChETA [127] have revealed that neuronal activity in the tumor microenvironment (TME) has a fundamental impact on tumor progression, especially in gliomas [43,44] and prostate cancer [44,128]. The TME is the immediate surrounding of a malignant tumor and, in addition to cancer cells and cancer stem cells, consists of nerve connections, innate and adaptive immune cells, fibroblasts, and endothelial cells [128]. The unique immunosuppressive, low pH and nutrient-poor environment, as well as bioelectrical mechanisms happening in the TME, can have a significant impact on tumorigenesis [51,128].

Optical stimulation of pyramidal excitatory neurons in the TME led to glioma growth and invasion [126]. Progressive tumor infiltration induced changes in the function of adjacent neuronal tissue [43] by creating a hyperexcitable bidirectional network between excitatory peritumoral neurons with synaptic glutamatergic AMPA receptors [43,126] and glioma cells [126], facilitating tumor growth [43,51] through calcium signaling [51]. Activity in excitable neuronal-glioma interfaces also regulated ion currents [51] and the secretion of mitogenic proteins like brain-derived neurotrophic factor (BDNF) and neuroligin-3 (NLGN-3), a molecule vital for neuronal plasticity and synaptic function [44]. NLGN-3 secretion [44] and pyramidal cell activity-induced membrane depolarization [43] in the TME created a positive feedforward loop in glioma progression [44] by activating ion channels [51]. By interacting with oncogenic signaling pathways, like PI3K/Akt/mTor, they continuously augmented oncogenic protein expression [44,51]. Also neuronal-circuit mechanisms following visual deprivation [126] and chronic stress [125] facilitated growth in gliomas [126] and breast cancers [125].

In contrast to the growth-promoting effects of excitatory glutamatergic neurons, optogenetic stimulation of inhibitory GABAergic parvalbumin interneurons attenuated tumor cell proliferation [126]. Furthermore, optogenetic control of incessant human glioma cell membrane depolarization through ChETA significantly decreased cell viability by decreasing cyclin expression, thus attenuating proliferation and inducing mitochondria-mediated apoptosis. In mouse experiments using intracranial optical fiber light stimulation, optogenetics selectively inhibited glioma growth, decreased tumor size, and prolonged survival rates [127]. In *Xenopus laevis*, a genetically encoded ChR2 variant enabled photocontrol over tumorigenesis through bioelectricity [40]. Hyperpolarization led to reduced activity of Kras, which is commonly overexpressed in tumors, thus counteracting tumorigenesis and subsequently leading to tumor regression in vivo.

Overall, a broad and still increasing range of cell signaling pathways with relevance for cancer development and progression has been investigated with a variety of optogenetic tools. Many of the earlier studies focused on the feasibility of bringing optical control to a specific pathway and often focused on showing that light control could confirm and complement what had previously been established with inducible expression systems or pharmacologic inhibitors. The true strength of optogenetics in this area lies, however, in temporal patterns like repeated short activation pulses or stimulation of cellular subcompartments that cannot be achieved to the same degree with other methods. Hence, optogenetics has contributed to a more dynamic picture of the fine-tuned processes underpinning, for instance, cell fate decisions or cell migration and invasion of cancer cells.

### 4.2. Modulating Immune Functions

**Figure 4:**
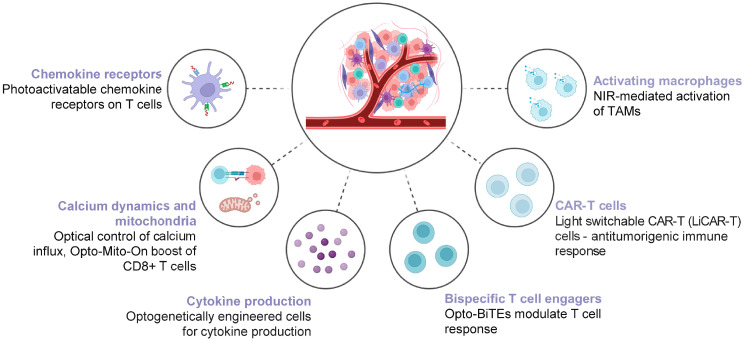
Examples of ways in which optogenetic tools have been used to modulate immune functions in model systems.

Cancer therapies based on activation of the immune system have an ever-increasing therapeutic impact [129]. Nevertheless, off-target effects and severe immune responses are common due to nonspecific targets of pharmacological cancer immunotherapies. Incorporating optogenetics into immune-engineering may provide the most effective form of immunotherapy. The ability to work synergistically with other forms of immunotherapy and additionally implement light-mediated control over immune cells may thus not only enhance precision but also improve efficacy and safety and could minimize the occurrence of severe systemic side effects [55,130]. Although deep light penetration sources for in vivo use and their potential toxicity are still an issue [55], the implementation of optogenetics, coupled with upconversion nanoparticles (UCNP) [17], LED chips [26,131], or hydrogel implants [132], offer potential solutions to enhance the efficacy of in vivo cancer immunotherapies [26,131]. An overview of different approaches for the light-mediated modulation of immune functions is presented in Figure 4.

#### 4.2.1. Chemokine Receptors

To increase the density of immune cells in the tumor tissue and TME, as well as to gain knowledge about the involved migration mechanisms, mouse T cells expressing a chemokine receptor photoactivatable with rhodopsin were generated [133]. These cells show phototaxis and migrate towards the tumor site in vivo in response to light signals, thereby augmenting adoptive cell transfer and inducing tumor rejection. This technology could be adapted to impart light sensitivity to other types of chemokine receptors that play a more direct role in meditating dendritic cell homing or T cell recruitment to specific tumor sites [130].

#### 4.2.2. Calcium Dynamics in T Cells

T lymphocyte function is governed by Ca^2+^ influx into the cells via Ca^2+^ release-activated Ca^2+^ (CRAC) channels [134]. This leads, for instance, to activation of NFAT (nuclear factor of activated T cells), subsequently resulting in expression of genes mediating immune responses, like cytokines or interleukins [26,130,131]. Optical control of calcium influx and subsequent signaling in cytotoxic T lymphocytes (CTLs) via Opto-CRAC [131], melanopsin [26], and CatCh [45] enables spatiotemporal control of cytolytic tumor cell killing and thereby overcoming TME-associated immunosuppression [26,45,131]. Optogenetics enables precise light-based control of gene circuit activation and causes only low-level off-target cytotoxicity of T cells, while exerting improved systemic anti-tumor effects [26,45,131]. Optogenetically engineered CTLs reduced tumor burden in melanoma [116] and hepatocellular carcinoma bearing mice [26] due to enhanced and more precise T cell toxicity mediated by Ca^2+^/NFAT-dependent cytokine production, ultimately leading to apoptosis of cancer cells [26]. Optical control over CRAC channels could also be utilized in chimeric antigen receptor CAR-T cell therapies to precisely control Ca^2+^/NFAT signaling. This approach has the potential to enhance the anti-tumor response of CAR-T cells in the TME [130].

#### 4.2.3. Function of Mitochondria

An optogenetic tool called Opto-Mito-On was shown to boost effector CD8+ T cell function, migration, and cytokine production by modulating mitochondrial membrane potential, thus increasing ATP production [135]. With such approaches, however, it must be considered for future clinical applications that excessive immune responses due to the elevation of cytokine levels in the blood could cause potentially fatal side effects [26].

#### 4.2.4. Cytokine Production

To circumvent this issue, local and temporal control of cytokine release is required. Human mesenchymal stem cells transfected with optogenetically engineered plasmids (far-red light-controlled immunomodulatory engineered cells, FLICs) were loaded onto hydrogel scaffold implants for precise cytokine production and delivery in vivo [132]. FLICs-loaded hydrogel implants executed anti-tumor immunity as they activated NK (natural killer) cells and CD8+ T cells. Moreover, they prompted a strong cessation of tumor growth and elicited T cell-mediated anti-tumor memory without excessive immune activation and signs of toxicity in mice. Hence, this optogenetics-based technology was able to protect against distal and local cancer reoccurrence at surgical sites after tumor resection.

#### 4.2.5. Bispecific T Cell Engagers (BiTEs)

BiTEs can direct T cell-mediated tumor cell killing after simultaneous binding to cancer cell antigens and T cell receptors [136]. Yet low half-life and rapid clearance require continuous infusion, which is time-consuming and expensive and may come with painful side effects for patients. Optogenetically engineered HEK293FT cells were used to secrete BiTEs against glypican 3 (GPC3) upon far-red light illumination [137]. The system was able to activate T cells to specifically eliminate GCP3-postive tumor cells. For long-term in vivo applications of this system, however, improvements in encapsulation of engineered cells are necessary to provide protection of the engineered cells against the immune system.

#### 4.2.6. CAR-T Cells

Immunotherapies with engineered CAR (chimeric antigen receptor)-T cells represent a breakthrough in the treatment of some hematologic malignancies, but still face potential safety issues arising from toxicity and excessive cytokine release [138]. Light-switchable CAR-T cells (LiCAR-T) generated with different light-sensitive proteins [25,111,139] enable spatiotemporal control of CAR-T cell activation and subsequent anti-tumorigenic immune responses, thus enhancing on-target precision and safety, while attenuating off-target cytotoxicity and reducing off-target cytokine release. LiCAR-T systems were shown to lead to cancer cell killing at selective tumor sites in melanoma [25,111] and lymphoma mouse models [25,111,139]. To overcome potential problems with high background CAR expression resulting in unwanted on-target off-tumor cytotoxicities, a system combining tamoxifen-mediated and light-mediated control of CAR expression was instated [65]. Only after priming with tamoxifen can light activate CAR expression preventing spontaneous activation and suppressing background activity.

Although some progress must be made for clinical applications, optogenetic systems can help to tailor personalized T cell-based therapies and limit toxicity by enhancing spatiotemporal control of CAR expression [25,26,131].

#### 4.2.7. Promoting Anti-Tumor Effects Through Dendritic Cells (DCs)

The STING (stimulator of interferon genes) pathway is a critical pathway for cancer immunotherapy [140]. The Cry2-based light-inducible LiSmore (Cry2) optogenetic system was demonstrated to be capable of activating the STING pathway and initiating subsequent cytokine production in dendritic cells (DCs), as well as evoking the activation of the innate and adaptive immune system [141]. In melanoma bearing mice, LiSmore in DCs facilitated dendritic cell maturation, promoted antigen presentation for effector T cell priming in tumor-draining lymph nodes, improved infiltration of cytotoxic lymphocytes (CTLs) into the TME, and demonstrated local and abscopal growth inhibition of tumors through enhanced CD8+ T cell cytotoxicity. In combination with a programmed death-ligand 1 (PD-L1) checkpoint inhibitor, LiSmore-DCs effectively attenuated tumor growth in LL/2 lung cancer mouse models, while promoting T cell memory and minimizing side effects through spatiotemporal control over anti-tumorigenic immunity. Control over calcium signaling via Opto-CRAC in dendritic cells is another approach that was shown to enhance cancer cell susceptibility to CTL-mediated killing and promote anti-tumor effects [131].

#### 4.2.8. Activating Macrophages

A plasmid composed of an interferon-gamma gene driven by heat shock protein 70 promoter was transfected into breast cancer cells. By employing conjugated polymer nanoparticles to convert NIR light into heat, NIR illumination resulted in pro-inflammatory cytokine expression and release, thus activating tumor-associated macrophages (TAMs). TAMs exhibited cytotoxicity towards cancer cells, reformed the immunosuppression of the TME, and upregulated major histocompatibility complexes (MHCs) facilitating adoptive immune responses [55].

In contrast to many studies dissecting signaling pathways, most studies investigating optogenetic immune cell modulation are aimed less at a better understanding of basic immune cell functions but more at improving control over specific immune cells with a translational perspective. Indeed several studies show improved tumor regression by the addition of the optogenetic approach as a proof of principle in animal models. While these are encouraging results, translation from mice to patients will be a challenging task.

### 4.3. Drug Discovery

By their ability to specifically and inexpensively activate a broad range of signaling pathways, optogenetic platforms are versatile tools for drug screening studies allowing an all-optical mode of operation by using light for both stimulus generation and readout of the signal [142]. Hence, the potential of optogenetics has been harnessed for drug discovery strategies in neuropharmacology [143,144] and cardiology [145,146] but also for screening of kinase inhibitors [147] and metabolically active agents [148] for anticancer applications [149]. Recent work has used optogenetics to identify modulators of cell stress response in a high-throughput screen [150]. In a different approach, the combination of light-activated Cre recombinase and a fluorescent Cre reporter was instrumental for inducing tumorigenesis in ex vivo mini-colons that represent a valuable tool for studying tumor initiation and screening for anticancer agents [151]. The recent description of a zebrafish strain with a light-activatable Cre recombinase for targeted expression of oncogenes will allow all-optical in vivo drug screens in zebrafish models [152,153].

Using optogenetics for drug development purposes holds considerable promise. It allows translating improved knowledge of tumor biology into benefits for cancer patients while avoiding the complexities and difficulties of direct applications of optogenetic constructs in patients. The recent developments of mini-colons and zebrafish models for that purpose offer promising new perspectives.

### 4.4. Killing Cancer Cells

Since the beginnings of cancer therapy, the ultimate, but often elusive, therapeutic goal has been the complete eradication of all cancerous cells from the body. Therefore, it is no surprise that also in the field of optogenetics multiple methods have been developed to directly or indirectly induce cancer cell death.

#### 4.4.1. Light-Mediated Induction of Regulated Cell Death

Several studies have used optogenetics to activate components of apoptotic and non-apoptotic cell death pathways. Fas-mediated apoptosis was induced by the light-controlled interaction of Fas with FADD (Fas-associated death domain protein) in an uveal melanoma model [154] and together with NIR-responsive UCNPs in HeLa xenografts [155]. The light-induced control of Fas/FADD interaction and its ability to trigger apoptosis was also used in a mouse model for optogenetic therapy of retinoblastoma [156]. To avoid the phototoxicity of external blue light for retinal cells, this model used nanoparticles camouflaged with macrophage membrane and in situ bioluminescence for activation of the system.

Induction of apoptosis was also observed after prolonged blue light illumination of ChR2 or its D156A variant in different cell models in vitro and in vivo [39,42]. The results of these studies suggest that chronic ChR2 activation causes Ca^2+^ overload in the cells and thereby triggers apoptosis [39,42]. Activation of channelrhodopsin expressed specifically in the inner mitochondrial membrane of cancer cells via bioluminescence from nanoluciferase expressed in the cytosol of the same cells was shown to trigger mitochondrial membrane depolarization and subsequent apoptosis of cancer cells in vivo [157]. Another recent approach has been the expression and green light activation of the outward proton pump archearhodopsing-3 resulting in apoptosis induction of xenotransplanted cancer cells through intracellular alkalinization [158].

Necroptosis and pyroptosis, two non-apoptotic forms of regulated cell death, have been brought under light-mediated control by the use of a set of optogenetic tools termed LiPOPs (light-induced non-apoptotic tools) using light-mediated oligomerization of the key signaling components receptor-interacting protein kinase 1 and 3 (RIPK1/3) and mixed lineage kinase domain-like protein (MLKL) for necroptosis and gasdermin D (GSDMD) for pyroptosis [159]. Together with UCNPs or bioluminescence, LiPOPs achieved optogenetic or chemo-optogenetic killing of cancer cells in several mouse cancer models.

#### 4.4.2. Optogenetics and Photodynamic Therapy

Photodynamic therapy (PDT) is a biomedical approach, in which reactive oxygen species (ROS) production upon illumination of photosensitizers in target cells triggers cell death [160]. Commonly used chemical photosensitizers, however, often show low biocompatibility and low therapeutic effects. As an additional approach, researchers have used ROS generating proteins (RGPs) such as KillerRed [161] or KillerOrange as genetically encoded photosensitizers [162]. These and another genetically encoded photosensitizer called miniSOG (mini Singlet Oxygen Generator) have been targeted to subcellular localizations like mitochondria, the plasma membrane, and chromatin and were shown to result in caspase-mediated cell death, DNA breaks, and senescence [161,163,164,165]. To further increase light penetration depth, UCNPs were used to activate KillerOrange in a HeLa xenograft mouse model [163]. After illumination of the accumulated photosensitizer in the mitochondria, ROS not only induced cancer cell apoptosis, but also the release of an siRNA enabling controllable gene therapy. This UCNP-coupled photosensitizer-based approach induced significant mitochondria-mediated apoptosis and consequently inhibited tumor growth in vivo.

#### 4.4.3. Optogenetically Engineered Bacteria and Viruses

Bacteria-mediated cancer therapy (BMCT) employs attenuated facultative anaerobic bacterial strains, since they can preferentially infiltrate tumor tissue and accumulate in the hypoxic and low pH TME, where they either directly kill cancer cells, activate the immune system, or release anticancer agents [166]. Different approaches have been used to add light-mediated control systems to BMCT. In one approach, an attenuated strain of Pseudomonas aeruginosa was engineered to switch between lifestyle modes (planktonic/biofilm/lysis) depending on the level of NIR illumination and thereby allow controlled tumor colonization and drug release [167]. *E. coli* strains were engineered to trigger cancer cell death by the light-controlled expression and release of cytolysin A (ClyA) [53], tumor necrosis factor alpha [23], or tumor necrosis factor alpha-related apoptosis inducing ligand (TRAIL) [168]. Also lactic acid bacteria (*L. lactis*) were shown to initiate cell death pathways in cancer cells after incorporation of plasmids designed for light-sensitive expression of interferon gamma [52,54]. Together with UCNPs for deep-tissue stimulation and in combination with other treatment modalities, these systems have demonstrated significant anti-tumor and anti-metastatic effects in mouse models and are considered as promising strategies for clinical applications.

Besides BMCT, oncolytic virotherapy aims to achieve immune system activation and tumor cell killing by tumor cell-selective virus replication [169]. Optogenetic technology was used to generate a photoactivatable oncolytic adenovirus (paOAd) that induces viral gene expression in human telomerase reverse transcriptase (hTERT)-positive human cancer cells upon blue light irradiation causing tumor cell death. In xenograft mouse models, paOAd substantially decreased cancer cell and cancer stem cell viability after blue light illumination [21].

While light-mediated induction of various forms of cancer cell death is an enticing perspective, translation to patients will face similar hurdles as optogenetic modulation of immune cells in patients. An additional strength of these approaches could be their use to decipher temporal or spatial stimulation patterns that most effectively trigger different modes of cell death and avoid resistance development.

## 5. Optogenetics and the Hallmark Traits of Cancer

**Figure 5:**
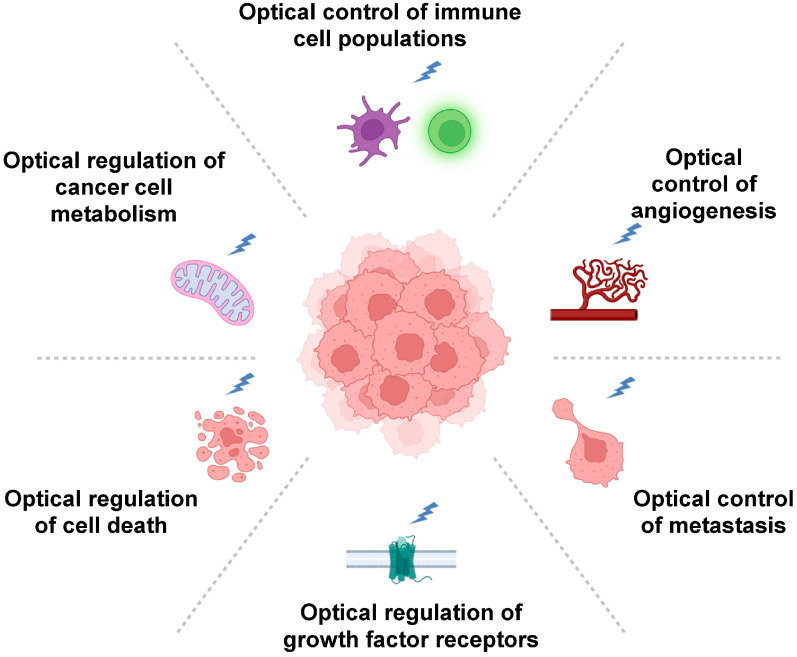
Overview of specific hallmark traits of cancer where optogenetic approaches have already made significant contributions for dissecting the dynamics of cancer development or for refining and improving control over therapeutic approaches.

Since its first description more than two decades ago, the concept that different types of cancer, irrespective of their cellular origin and specific mutations, are destined to acquire a set of distinct biological traits—termed cancer hallmarks—has been extremely useful for dissecting the process of cancer development [80,170,171]. Importantly, hallmark traits also present potential vulnerabilities of cancer that can be exploited for therapeutic interventions [80,172]. With their ability to control cellular processes remotely and with high spatial and temporal resolution, optogenetics—as outlined in the sections above—has not only offered innovative approaches to interrogate signaling networks controlling specific hallmark traits, but also enables disruption of cancer-driving circuits in more specific and less invasive ways. A lot of effort in the last decade has gone into the development and proof-of-principle demonstrations of individual optogenetic tools that are suitable for the investigation of specific hallmark traits. Not all cancer hallmarks, however, have received the same degree of attention. While multiple studies have applied optogenetics to investigate aspects of sustained proliferative signaling [94,173], invasion and metastasis [99,120], or deregulated cellular energetics [123,124], investigations of, for instance, replicative immortality are still missing. Figure 5 provides an overview of selected hallmark traits of cancer that have been investigated by optogenetics.

After more than a decade of application of optogenetic tools to cancer research, the question arises how this has impacted our view of cancer. Besides the elegant engineering approaches for combining optogenetics with immunotherapies that may prove at some point their value in the clinic, perhaps the most fundamental addition brought about by optogenetics is the heightened awareness of the temporal dimensions of cell signaling outcomes. Not just the simple on or off state of a signal but the temporal patterns of activation determine transmission and in consequence cell fate [173]. This has important implications for the interpretation of oncogenic mutations and their targeting with inhibitors that we are still just beginning to understand [94]. Although not a hallmark trait of cancer, the interconnectedness of neuronal activity and cancer growth is another important area fundamentally influenced by the addition of optogenetic approaches to the cancer field [43,44].

## 6. Remaining Challenges and Future Perspectives

While spatial and temporal precision are key strengths of optogenetic technologies and have been instrumental for dissecting the dynamics of signal transduction pathways, their application in animal models or patients often requires, in addition, activation in various tissues such as lungs, livers, or the digestive tract. The majority of existing light-sensitive proteins relies on activation spectra concentrated around 400–500 nm with significantly restricted tissue penetration depth, thereby substantially limiting optogenetics’ applicability in vivo [130]. Activation methods for optogenetics to control cellular functions in deep tissues include the insertion of fiber optic probes and implantation of micro LEDs [174]. These methods, however, can give rise to inherent issues like infection, inflammation, tissue damage, and unwanted immunologic reactions.

In comparison to visible light, which has only a tissue penetration depth of <1, 1–3, and 3–5 mm for blue, yellow/green, and red light, respectively, NIR light with a wavelength range of 700 to 1000 nm offers significantly deeper tissue penetration of 1–3 cm and induces less photodamage to mammalian cells due to less absorption and scattering within tissues [17,130,175]. Considering these advantages, there has been considerable effort to develop NIR-responsive molecules such as lanthanide-doped UCNP and photothermal absorbers with the capacity of activating optogenetic modules [175]. UCNPs possess the capability to convert NIR light into visible light with a lower wavelength, thus facilitating the activation of a variety of light-sensitive protein domains used in optogenetics. The customizability of emission wavelengths of UCNPs are key advantages of UCNPs and make them stand out as the most promising and versatile NIR-responsive tool [17,175]. However, with respect to clinical applications, safety concerns arising from the inorganic origin of UCNPs exist, particularly regarding their long-term safety. A comprehensive assessment of the toxicity potentially caused by UCNPs’ surface modifications, size, and chemical composition across various tissues and organs is required. Moreover, the limited emission strength and the absence of specific cell-type targeting capabilities of most UCNPs remain limitations for their application. Furthermore, precise control of laser power density and exposure time are crucial to mitigate potential damaging side effects associated with photothermal overheating. Directly NIR-activated optogenetic tools such as iLight or MagRed present alternatives for deep-tissue activation [176,177]. For applications beyond the reach of NIR, activation of optogenetic proteins by bioluminescence from nanoluciferase [156,157] may be an alternative to the insertion of optical fibers, but will add complexity and reduce spatial and temporal control.

Basal activity of optogenetic constructs in the absence of light or unwanted activation by ambient light or light scattering in non-target cells are of concern for application of optogenetics in therapeutic settings. The use of promoters that result in expression only in target cells may limit such problems [178]. The dynamic range of a light-controlled response is of special relevance, for instance, for the expression of a therapeutic gene, but may be less relevant for more binary outcomes such as light-mediated gene editing.

A further challenge in clinical application is the task of identifying appropriate chassis cells for introducing synthetic optogenetic circuits into humans without adverse immunogenic side effects. Prospective clinical applications may necessitate the use of autologous cells, for example, patients’ own mesenchymal stem cells, a method that has already been clinically validated [179]. Immunogenicity or cytotoxicity may, however, also result from the expression of the optogenetic proteins themselves, especially as they often contain non-human sequences. While potentially helpful in clinical scenarios in which elimination of cancer cells is intended, this may severely hamper efficacy in cases where the prolonged presence of cells expressing the optogenetic proteins is required. Humanization of the protein and administration of immunosuppressants may be useful strategies in such cases. Moreover, as with gene therapy approaches in general, mutations in genes of the target cells generated by the insertion of the construct into the host cell genome (insertional mutagenesis) is a critical issue that needs to be considered [180].

A key issue in all targeting approaches for cancer is the question how many of the target cells can be reached. Adeno-associated viruses (AAV) are often used for gene therapy approaches due to their minimal pathogenicity and ability for long-term transgene expression without integrating into the host genome [181]. Even the best vector design, however, is unlikely to allow expressing an optogenetic construct in all cells of a tumor. Thus, only approaches that generate a strong enough bystander effect (the ability to also kill nearby cancer cells not expressing the construct via a diffusible toxic product such as ROS) or approaches that harness the power of the immune system to generate a systemic response will be able to generate longer-lasting anticancer effects [130,182].

The existing literature highlights the versatility of optogenetics in probing intricate cellular processes. Hence, optogenetics emerges as a key tool to gain knowledge about intracellular processes involved in signal transduction associated with oncogenesis and metastasis. It is exactly this knowledge about key dependencies within signaling networks that can turn hallmark traits into vulnerabilities of cancer and enables rational and precise therapeutic interference. The expansion of optogenetic tools that has taken place over recent years increasingly enables multiplexing of tools with different spectral sensitivities and thereby vastly increasing the possibilities for combinatorial and sequential activation or interruption of pathways. In many cases, pharmacology will be able to provide inhibitors for targeting biomolecules identified by optogenetics as critical for the progression and metastasis of specific cancers. The direct application of optogenetics in clinical trials has so far been limited to vision restoration [183]. For expansion into clinical trials in the oncology field especially the potential for combination with gene therapy [20], bacteria- or virus-mediated therapies [20,22], photodynamic therapy [163], and, in particular immunotherapies [25] are exiting future perspectives.

## 7. Conclusions

The last few years have seen a vast expansion of optogenetic tools and approaches than can be—and have been—applied to questions in cancer research. They increasingly allow a more dynamic analysis of the behavior of cancer cells and their interactions with the tumor microenvironment. This increased knowledge can be translated into benefits for patients by informing pharmacologic treatment strategies and facilitating drug screening. Strategies for direct applications in cancer patients have been envisioned, but still need to overcome considerable challenges.

## Figures and Tables

**Table 1:** Source organisms, modes of action, activation and reversion wavelengths, and temporal performances of selected photoreceptors used in optogenetics.

Light-Sensitive Protein Domain	Source Organism	Mode of Action	Excitation Wavelength (nm)	Excitation Kinetic	Reference
UVR8	*Arabidopsis* *thaliana*	Heterodimerization with COP1, homodimerization	300 nm Reversion: dark	Milliseconds Reversion: hours	[27]
PhoCl	*Clavularia* sp.	Photocleavage	380 nm Reversion: -	Minutes Reversion: -	[28]
CRY2 (Cryptochrome)	*Arabidopsis* *thaliana*	Heterodimerization with CIB1, oligomerization	450 nm Reversion: dark	Seconds Reversion: minutes	[29]
ASLOV2 (LOV Domain)	*Avena sativa*	Intramolecular conformation change	450 nm Reversion: dark	Seconds Reversion: tens of seconds	[30]
mOpn4L (Melanopsin)	*Mus musculus*	G-protein-coupled receptor activation	480 nm Reversion: 560 nm	Seconds Reversion: tens of seconds	[31]
ChR2 (Channelrhodopsin)	*Chlamydomonas reinhardtii*	Opening of cation-selective channel	450 nm Reversion: dark	Milliseconds Reversion: milliseconds	[32]
NpHR (Halorhodopsin)	*Natromonas pharaonis*	Opening of chloride channel	590 nm Reversion: dark	Milliseconds Reversion: tens of milliseconds	[33]
PhyB (Phytochrome)	*Arabidopsis* *thaliana*	Heterodimerization with PIF3/6	660 nm Reversion: 740 nm	Milliseconds Reversion: milliseconds	[34]
BphP1 (Phytochrome)	*Rhodopseudo-monas palustris*	Heterodimerization with PpsR2 or Q-PAS1	760 nm Reversion: 640 nm	Seconds Reversion: seconds	[35]

## Data Availability

No new data were created or analyzed in this study. Data sharing is not applicable to this article.

## Abbreviations

AMPK	Adenosine monophosphate-activated protein kinase
ATP	Adenosine triphosphate
BiTEs	Bispecific T cell Engagers
BMCP	Bacteria-mediated cancer therapy
BMP	Bone morphogenetic protein
BR	Bacteriorhodopsin
CAR	Chimeric antigen receptor
Cas	CRISPR-associated protein
CCE	Constitutive calcium entry
ChR	Channelrhodopsin
CRAC	Ca^2+^ release-activated Ca^2+^
CRISPR	Clustered Regularly Interspaced Palindromic Repeat
CRY	Cryptochrome
CTL	Cytotoxic T lymphocyte
DC	Dendritic cell
EGFR	Epidermal growth factor receptor
EMT	Epithelial-to-mesenchymal transition
Erk	Extracellular signal-regulated kinase
FADD	Fas-associated death domain protein
FGFR	Fibroblast growth factor receptor
FLIC	Far-red light-controlled immunomodulatory engineered cells
GDF	Growth and differentiation factor
GEF	Guanine nucleotide exchange factor
GPC3	Glypican 3
GPX4	Glutathione peroxidase 4
HEK	Human embryonic kidney
HR	Halorhodopsin
HSP70	Heat shock protein 70
LED	Light-emitting diode
LOV	Light–oxygen–voltage
MAPK	Mitogen-activated protein kinase
MHC	Major histocompatibility complex
mTOR	Mammalian target of rapamycin
NFAT	Nuclear factor of activated T cells
NIR	Near-infrared
NK	Natural killer
NLGN	Neuroligin
NLS	Nuclear localization signal
PI3K	Phosphoinositide 3-kinase
PHY	Phytochrome
PK	Pyruvate kinase
PTD	Photodynamic therapy
RET	Rearranged during transformation
ROS	Reactive oxygen species
RTK	Receptor tyrosine kinase
SOS	Son of sevenless
STING	Stimulator of interferon genes
TAM	Tumor-associated macrophage
TGF	Transforming growth factor
TME	Tumor microenvironment
UCNP	Upconversion nanoparticles
VEGFR	Vascular endothelial growth factor receptor
